# Successful Non-Microsurgical Partial Ear ‘Spare Parts Reconstruction’ After 24 Hours — A Case Report

**DOI:** 10.1055/s-0045-1814751

**Published:** 2026-02-06

**Authors:** Girish Mirajkar, Sushrut A. Raut, Amit Peswani, Abhishek Dhakad, Sahil Waghmare, Richa Goel, Tulika Agrawal, Uday Bhat

**Affiliations:** 1 Department of Plastic Surgery, Topiwala National Medical College and B.Y.L. Nair Charitable Hospital, Mumbai, Maharashtra, India; 2 Department of Plastic Surgery, Vitthal Sayanna General Hospital, Thane, Maharashtra, India; 3 Department of Plastic Surgery, ESIC Hospital, Nashik, Maharashtra, India

**Keywords:** auricle reconstruction, ear amputation, human ear-bite, Baudet's technique ear reconstruction, Mladick's modification

## Abstract

Traumatic ear amputation secondary to human ear-bite is one of the common emergencies. If left untreated, it results in a deformity that causes social embarrassment and stigmatizes the victim. This case study documents primary ear reconstruction with “spare-part” amputated cartilage in a middle-aged, diabetic male after 24 hours of amputation. This reconstruction was performed using modified Mladick's Pocket technique in three stages over 90 days. There was complete cartilage take. Partial flap necrosis was managed conservatively. Over 18 months, the ear settled well and was nearly symmetrical to the opposite ear. There was a good skin match and minimal donor site morbidity. In favorable circumstances, primary non-microsurgical reconstruction using amputated cartilage of the ear in human bite is a feasible option even after 24 hours of amputation in patients with comorbidities. Primary ear reconstruction has the technical advantage of having unscarred tissues for repair.

## Introduction

Delayed presentation of traumatic ear amputation secondary to human ear-bite is a frequent presentation in tertiary hospitals in India. Although the wound heals secondarily if left untreated, it results in deformity of the ear, which causes social embarrassment to the patient. Hence, it becomes imperative to take cognizance of this problem and consider therapeutic activism in the form of replantation whenever feasible. But a clinical dilemma arises when the patient appears well beyond the generally accepted golden period of 4 to 6 hours.

In this case report, we present a case of primary non-microsurgical spare part reconstruction by using the amputated cartilage of the helix and antihelix of the left ear in a middle-aged, diabetic male, 24 hours after the injury.

## Case Report

**Fig. 1:**
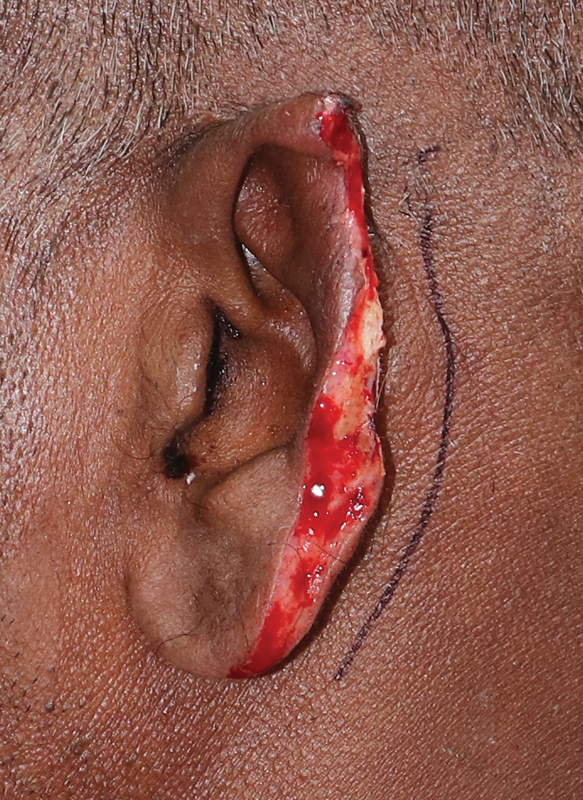
Figure showing the clinical presentation of the patient showing left ear helix and antihelix amputation.

**Fig. 2:**
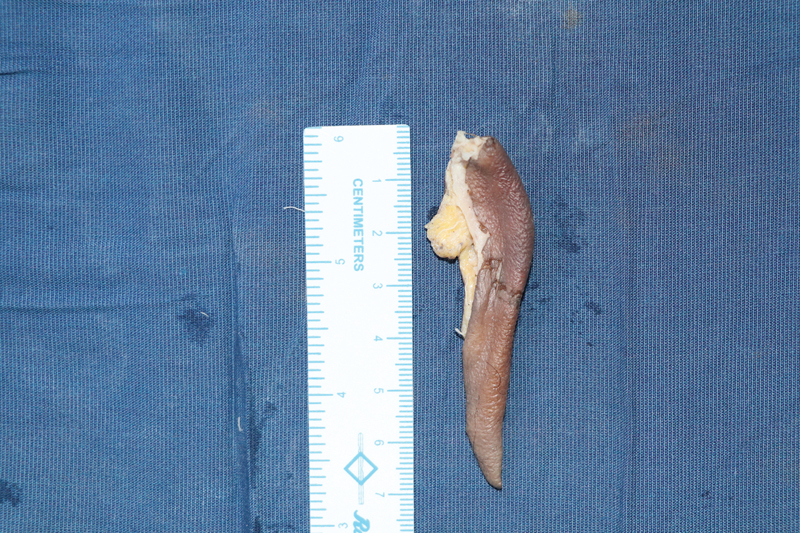
Figure showing the amputated helix and antihelix. It is relatively clean and shows a dusky hue.

A 52-year-old male rickshaw driver got his left ear completely bitten off by a person he was quarrelling with. He presented 23 hours after the incident. The patient was vitally stable without any known comorbidities. There were no other injuries. The right ear was found to be normal. The left ear helix and partial antihelix were absent ( Fig. 1 ). The patient's relatives had brought the amputated ear preserved in a plastic bag covered with an ice pack. It was not grossly contaminated, but the skin showed a dusky hue, as shown in Fig. 2 . During his hospital stay, he was diagnosed with type II diabetes mellitus.

By the time the patient was optimized and taken up for surgery, it was 25 hours since the amputation. Our plan was Mladick's technique of ear reconstruction 1 using the cartilage of the amputated ear, with a slight change considering our patient profile. It was done in the following stages:

### Stage 1

**Fig. 3:**
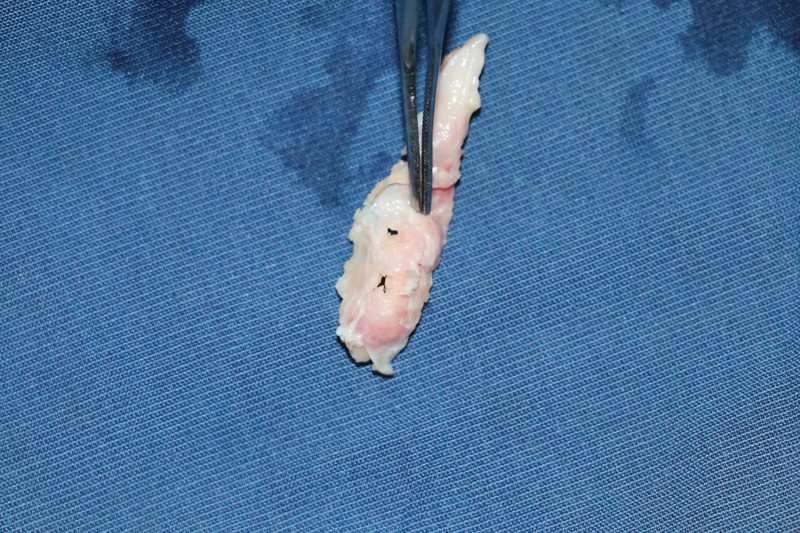
Figure showing fenestration of the degloved amputated cartilage.

The amputated ear was washed with dilute betadine. The cartilage was dissected free from the skin and fenestrated at multiple points, and the skin was discarded ( Fig. 3 ). This cartilage was then placed in a solution of triple antibiotic (ceftriaxone, metronidazole, and amikacin) for a period of 30 minutes.

**Fig. 4:**
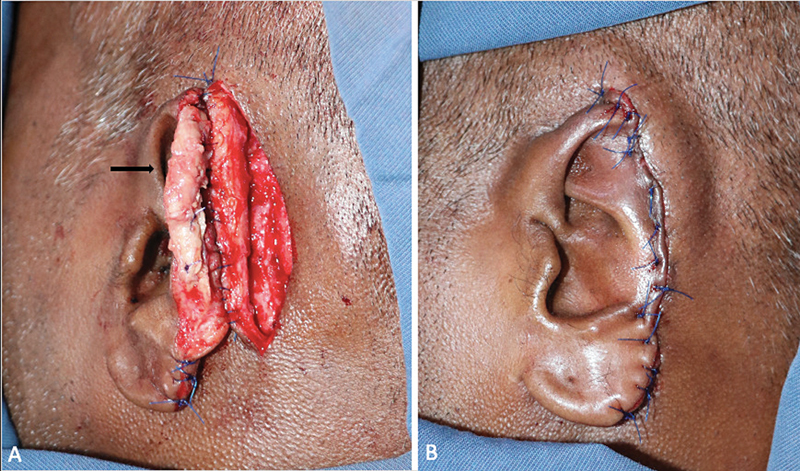
Stage 1: Reconstruction ( **A** ) showing fixation of the amputated cartilage to the residual ear, ( **B** ) replanted cartilage covered by the retroauricular flap at the end of stage 1.

The great auricular and retroauricular nerve block was given. The incision for creating a retroauricular tunnel was marked corresponding to the amputated residual ear ( Fig. 1 ). The medial edge of the ear stump was freshened and then sutured to the anterior edge of the tunnel. After thoroughly washing the native ear surface, the fenestrated cartilage was sutured to the intact cartilage with the help of polydioxanone 5–0 by horizontal mattress sutures ( Fig. 4A ).

The lateral border of the ear was sutured with the posterior edge of the retroauricular tunnel, thereby completely covering the replanted cartilage ( Fig. 4B ). The skin was sutured with polypropylene 4–0 suture. The patient was given antibiotic coverage for aerobic and anaerobic organisms. Management of diabetes was done with endocrinology assistance.

### Stage 2 (after 4 weeks)

**Fig. 5:**
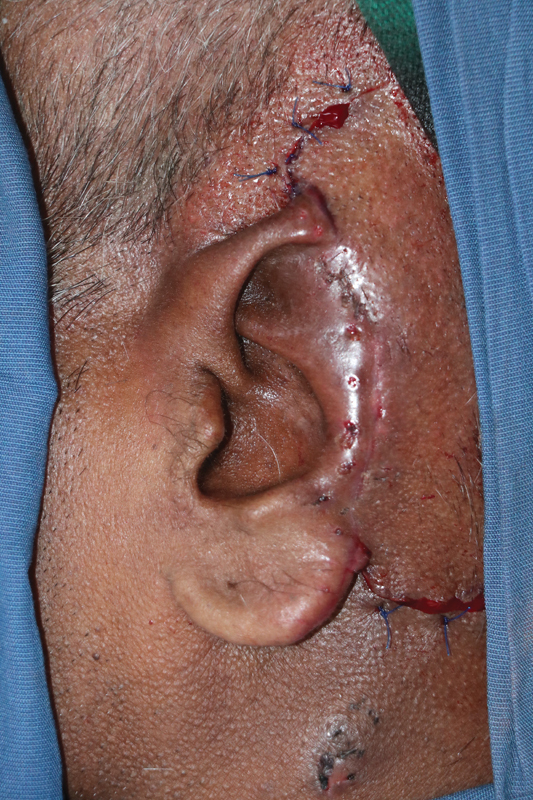
Figure shows a delay being done for the final flap design.

We performed a delay for the planned mastoid skin flap by placing 2 cm horizontal incisions superiorly and inferiorly that were resutured with polypropylene 4–0 suture ( Fig. 5 ).

### Stage 3: Retroauricular Flap Raised and Inset (after 4 weeks of Stage 2)

**Fig. 6:**
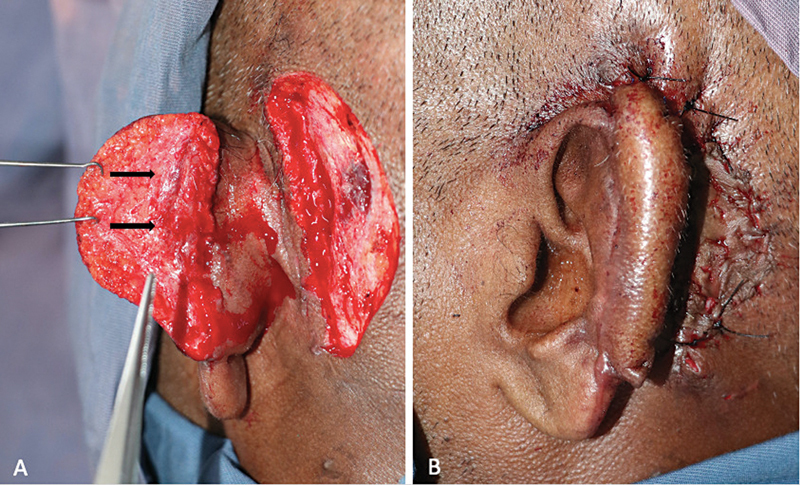
( **A** ) Figure showing raised retroauricular flap with the replanted cartilage with complete take. ( **B** ) shows inset of the flap after folding over itself.

The estimated skin requirement was measured by using the opposite ear as a template. This template was reversed and used for marking the skin over the retroauricular tunnel over the mastoid region. After raising the flap, we could appreciate complete take of the replanted cartilage that maintained its shape ( Fig. 6A ). The sutured posterior edge of the ear to the anterior edge of the tunnel was taken down. The flap of the tunnel created was then wrapped around the cartilage and sutured with the posterior edge of the ear ( Fig. 6B ).

**Fig. 7:**
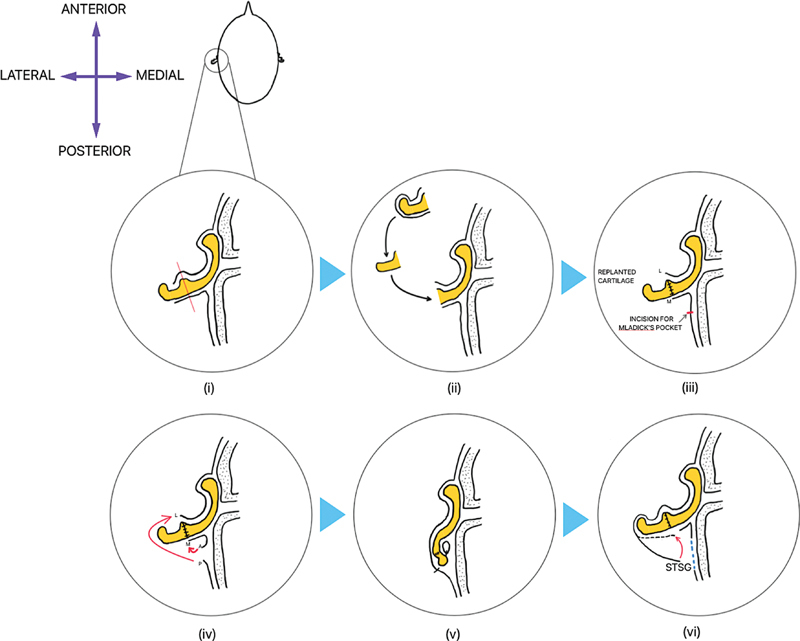
Schematic representation of the entire reconstruction of the left ear in axial section. (i) shows the amputation of the ear. (ii) shows degloved cartilage being sutured to the native ear cartilage in anatomic position. (iii, iv) show sutured cartilage and creation of the retroauricular tunnel. M is the medial edge of the ear. L is the lateral edge of the ear. A is the anterior edge of the retroauricular tunnel, and P is the posterior edge of the tunnel. (v) Replanted cartilage having vascular cover at the end of the first stage. (vi) shows the third stage in which the flap is raised based on the lateral edge of the ear. It is folded on itself, and the posterior edge of this flap is sutured to the medial edge of the cut end of the ear. The donor site of the flap is covered with a split-thickness skin graft (STSG).

The steps of the surgery performed are summarized in a schematic form in Fig. 7.

The patient was followed up serially at 3, 6, and 18 months and serial photography was done to observe the changes in the skin flap color and shape of replanted cartilage.

## Results

On day 3, the posterosuperior edge of the flap started showing discoloration. Superiorly, a few sutures were removed to relieve the tension secondary to edema, but managed conservatively with a foam dressing. The necrosis was full-thickness and did not advance. It was approximately 2 cm ^2^ in size and left undisturbed. This formed an eschar that allowed secondary healing underneath. Over a period of 3 weeks, the flap healed with secondary intention as the eschar came off spontaneously.

**Fig. 8:**
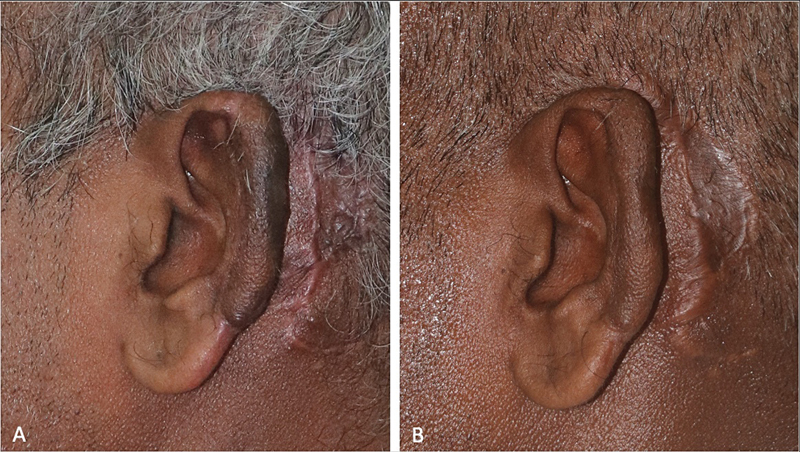
( **A** ) Shows reconstructed ear at 6 months, and ( **B** ) shows the reconstructed ear at 18 months. It shows a near-symmetrical projection and height. At 18 months, hyperpigmentation had reduced, and the color match was satisfactory.

At 6 months, the flap had settled. The projection and height were symmetrical. There was hyperpigmentation of the flap skin. Cartilage had reasonable pliability ( Fig. 8A ).

**Fig. 9:**
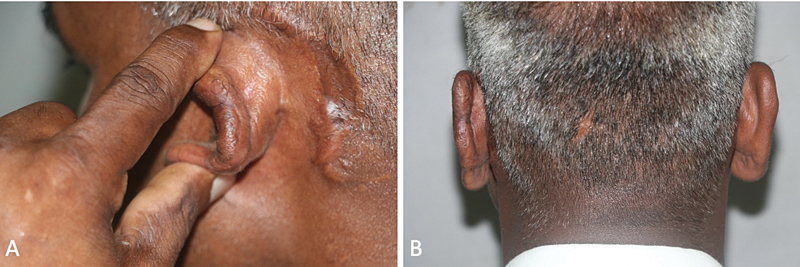
Figure showing good pliability of the ear ( **A** ) and symmetrical projection ( **B** ) at 18 months.

At 18 months, the flap color match was good with pliability as good as the normal ear, and the patient was satisfied ( Fig. 8B ). The reconstructed ear had good pliability and reasonable symmetry as shown in Fig. 9 .

## Discussion and Review of Literature

Secondary reconstruction of the ear is fraught with the difficult dissection of the recipient ear due to a lack of tissue planes and fibrosis. Additionally, outcomes are not as good as replantation. Waiting for reconstruction with deformity is embarrassing for the patient.

Microsurgical replantation is considered the gold standard since Pennington et al 2 performed the first successful replantation. The challenges with microsurgical reconstruction lie with the organization of the team, equipment, and expertise. Since 1980, many cases of microvascular replants of the ear have been reported with good success. In the absence of ideal conditions for microsurgical replantation, the use of the amputated cartilage as a free graft has been used to reconstruct the ear by replantation using surrounding tissue for vascular cover within a few hours of amputation.

The first such technique described was Mladick's pocket technique (1971)—the cartilage is degloved and then replanted to the amputated ear, which is then buried in the postauricular pocket. This pocket thereby provides nourishment to the replanted cartilage via the new channel developing between the temporoparietal fascia supplied by the superficial temporal artery and the deep branch of the middle temporal artery. In the second stage, the cover is fashioned out of the flap created from the postauricular skin.

Baudet et al, in 1971, 3 described replantation of the ear with intact anterior skin but degloved posteriorly over the pocket created posteriorly. The dermis is left intact to help promote later re-epithelialization and reduce cartilage warping. Also, intact skin helps prevent infection. This technique may result in the de-epithelialization over the anterior surface of the auricle or loss of skin over the epithelium.

Mladick and Carraway 4 revised their own technique in 1973, where the cartilage was replanted after dermabrasion of the ear epidermis instead of removing the whole skin thickness. It is then covered with the help of the postauricular pocket. The second stage remains the same as that of the original technique. This helps prevent flattening of the cartilage by the draped skin.

Park et al 5 introduced an alternative method for burying amputated auricular cartilage, which involves preserving skin only over the helix while removing it from the rest of the graft. In this approach, the exposed cartilage is placed between a retroauricular flap laterally and a facial flap medially. Despite its utility, the exposed helical skin may become necrotic, and the procedure necessitates three separate stages to yield a satisfactory outcome. A comparable technique was suggested by Destro and Speranzini, 6 where the skin is retained solely over the conchal area, with the rest being removed. The cartilage is then perforated at multiple sites and covered using a postauricular flap. Elevation of the ear is performed in a subsequent surgical stage.

For more extensive trauma involving loss of skin in the auricular region, some authors have recommended using a platysma musculocutaneous flap. de Mello-Filho et al 7 described a technique in which the amputated auricular cartilage is implanted into the platysma muscle and subsequently transferred back to its original position as a combined musculocutaneous–cartilaginous flap. Additionally, other authors have proposed reconstructing partial or complete traumatic auricular defects using a free flap harvested from the opposite ear. However, these methods rely on microsurgical techniques, which come with previously noted limitations.

In our case, since more than 24 hours had passed, we were tempted to bank the cartilage in the abdomen in the subcutaneous plane, such that secondary reconstruction could be done. Clean-cut edges and the absence of crushed elements encouraged us to attempt replantation of the cartilage. The only modification of the technique described by Mladick was adding a stage of delay—considering diabetic microangiopathy. 1 Despite the prolonged ischemia, the complete take of the graft was visualized.

The limitation of this technique lies in the clinical judgement of the condition of the tissues to be used. Failure of this technique can make further reconstruction using skin over the mastoid difficult. Although skin in the retroauricular sulcus can be used.

## Conclusion

Non-microsurgical replantation must be considered as the first choice even in cases with delayed presentation of ear amputation (i.e., after 24 hours), provided the amputated ear is clean cut, minimally contaminated, and vessels not fit for microsurgical replantation.
